# Pyrrolobenzodiazepines (PBDs) Do Not Bind to DNA G-Quadruplexes

**DOI:** 10.1371/journal.pone.0105021

**Published:** 2014-08-18

**Authors:** Khondaker M. Rahman, David B. Corcoran, Tam T. T. Bui, Paul J. M. Jackson, David E. Thurston

**Affiliations:** Department of Pharmacy, Institute of Pharmaceutical Science, King's College London, London, United Kingdom; University of Quebect at Trois-Rivieres, Canada

## Abstract

The pyrrolo[2,1-*c*][1,4] benzodiazepines (PBDs) are a family of sequence-selective, minor-groove binding DNA-interactive agents that covalently attach to guanine residues. A recent publication in *this journal* (Raju *et al*, *PloS One*, 2012, 7, 4, e35920) reported that two PBD molecules were observed to bind with high affinity to the telomeric quadruplex of *Tetrahymena glaucoma* based on Electrospray Ionisation Mass Spectrometry (ESI-MS), Circular Dichroism, UV-Visible and Fluorescence spectroscopy data. This was a surprising result given the close 3-dimensional shape match between the structure of all PBD molecules and the minor groove of duplex DNA, and the completely different 3-dimensional structure of quadruplex DNA. Therefore, we evaluated the interaction of eight PBD molecules of diverse structure with a range of parallel, antiparallel and mixed DNA quadruplexes using DNA Thermal Denaturation, Circular Dichroism and Molecular Dynamics Simulations. Those PBD molecules without large C8-substitutents had an insignificant affinity for the eight quadruplex types, although those with large π-system-containing C8-substituents (as with the compounds evaluated by Raju and co-workers) were found to interact to some extent. Our molecular dynamics simulations support the likelihood that molecules of this type, including those examined by Raju and co-workers, interact with quadruplex DNA through their C8-substituents rather than the PBD moiety itself. It is important for the literature to be clear on this matter, as the mechanism of action of these agents will be under close scrutiny in the near future due to the growing number of PBD-based agents entering the clinic as both single-agents and as components of antibody-drug conjugates (ADCs).

## Introduction

**Figure 1:**
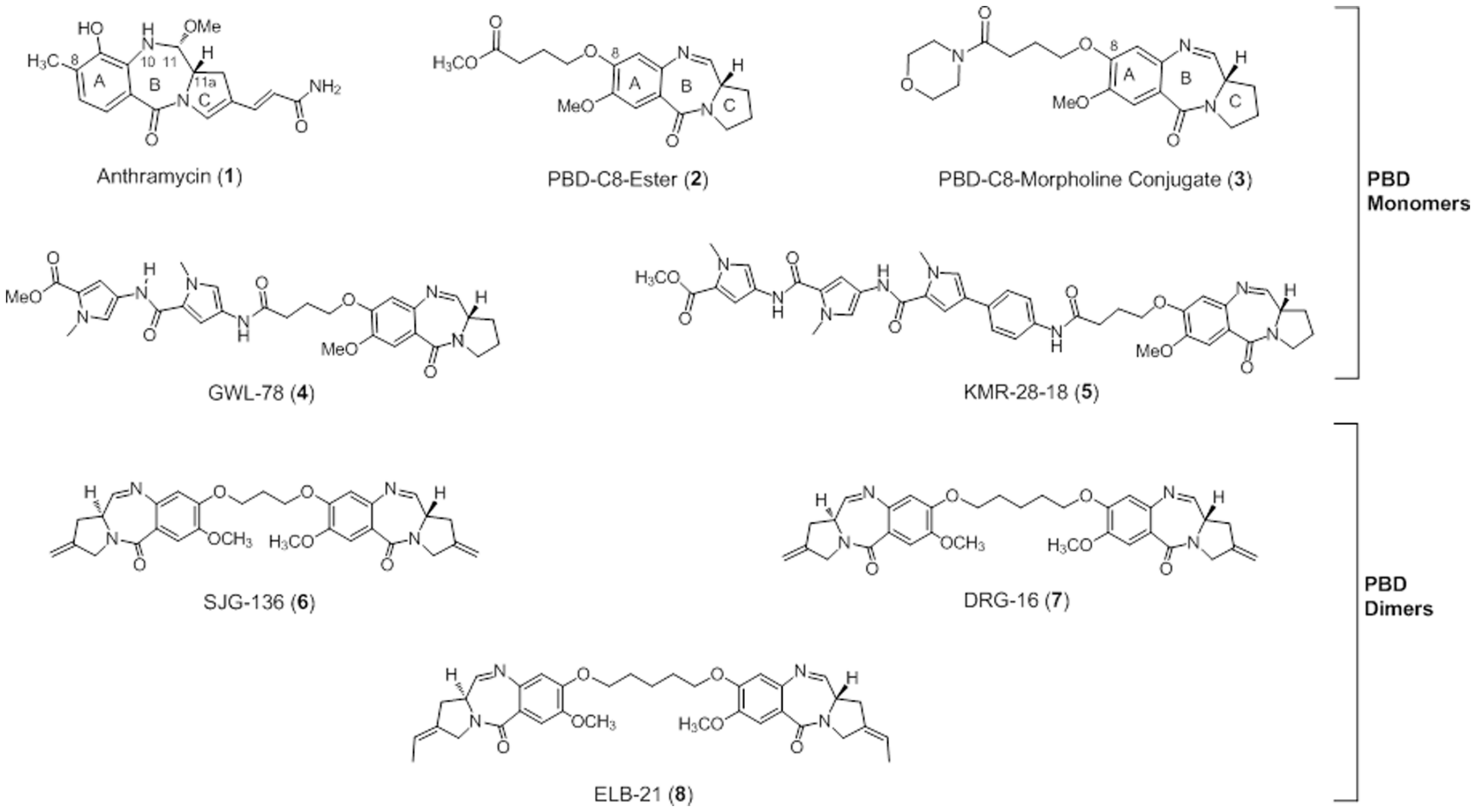
Structures of the PBD monomers and dimers used in the current study. Anthramycin methyl ether (**1**), the PBD-C8-ester (**2**) and the PBD-C8-morpholine (**3**) do not possess any π-systems in their C8-side chains, whereas the PBD C8-conjugates **4** and **5** both have C8-substituents consisting of aromatic rings. The three PBD dimers, SJG-136 (**6**), DRG-16 (**7**) and ELB-21 (**8**) have structural similarity to the PBD dimer (PBD2, **10**) evaluated by Raju and co-workers [53] (see **Figure 2**).

The pyrrolo[2,1-*c*][1,4] benzodiazepines (PBDs) are a group of sequence-selective DNA minor-groove binding agents originally discovered in *Streptomyces* species [1], [2], [3], [4], [5]. Anthramycin (**1**, **Figure 1**) was the first PBD to be isolated and studied [6], although more than twelve naturally occurring PBDs are now known [1]. They are characterized by an electrophilic N10–C11 imine group (or the hydrated equivalent) which forms a reversible covalent aminal linkage from their C11-position to the C2-NH_2_ group of a guanine in the DNA minor groove [7], [8]. Crucially, the molecules have (*S*)-chirality at their C11a-position, and this provides them with the appropriate 3-dimensional shape (*i.e.*, isohelicity) to fit perfectly into the DNA minor groove. PBD/DNA adduct formation has been shown to inhibit a number of biological processes, including the binding of transcription factors to DNA [9], [10], [11], [12] and the function of enzymes such as endonucleases [13], [14] and RNA polymerase [15]. Many PBD molecules also have significant antimicrobial activity [16], [17], [18], [19], [20], [21].

PBD monomers have been shown by footprinting [7], NMR [22], [23], molecular modeling [24] and X-ray crystallography [25] to span three base pairs with a reported preference for orientation of their A-ring toward the 3′-end of the covalently-modified strand (*i.e.*, A-Ring-3′) [26], although a recent report suggests that the A-ring can orientate toward either the 3′- or 5′-ends [27]. In the past, PBD molecules have been reported to prefer 5′-Pu-G-Pu-3′sequences [26], [28], although more recent data suggest that they have a kinetic preference for 5′-Py-G-Py-3′ motifs [27] (where Pu  =  purine, Py  =  pyrimidine, G  =  reacting guanine). PBDs are thought to interact with DNA by first locating a low-energy binding site through van der Waals (vdW), H-bonding and electrostatic interactions [29]. Once in place, nucleophilic attack by the exocyclic C2-NH_2_ of the central guanine then occurs to form a covalent adduct [29]. Once bound, the PBD remains anchored in the DNA host minor groove, avoiding DNA repair through negligible distortion of the helix [25]. It is well-established that PBDs are highly selective in requiring minor groove structure in either duplex or hairpin DNA for covalent binding to occur, and they do not bind to single stranded DNA (or RNA) regardless of its GC content and/or length [30].

Synthetic PBD monomers with non-covalent DNA-interactive components joined to the C8-position of their A-rings have also been reported [29], [31], [32], [33], [34], and examples such as GWL-78 (**4**, **Figure 1**) [29] and KMR-28-18 (**5**, **Figure 1**) [12] can span up to six or seven base pairs. Furthermore, two PBD units have been joined through their C7- [35] or C8-positions (*i.e.*, **6**-**8**, **Figure 1**) to form PBD dimers with the ability to form inter- and intra-strand DNA cross-links [36], [37]. One example (SJG-136, **6**) [38] is presently in Phase II clinical trials as an anti-cancer agent [39], [40], [41]. Both PBD monomers and dimers have a common feature in that their covalent interaction in the minor groove significantly enhances the stability of the DNA double helix to thermal denaturation [26], [29], [42], and this stabilizing property correlates well with their *in vitro* cytotoxicity [29], [38], [42].

Although B-Form DNA is the predominant type present in cells, it is now known that single-stranded guanine-rich nucleic acid sequences can fold into four-stranded structures comprised of stacked tetrads formed by Hoogsteen hydrogen bonding of four guanines, stabilized overall by monovalent cations such as K^+^. These structures, known as G-quadruplexes (or G4s), are classified by their sequence, stoichiometry, polarity of each strand, how the loops connect the different strands, and the conformation of guanine glycosidic angles. For example, G-quadruplexes can be classified as uni-, bi- or tetra-molecular, and can have differences in loop directionality (*e.g.*, parallel or antiparallel) and length. Certain proteins known as telomere end-binding proteins (TEBPs) can promote G-quadruplex formation under the control of the cell cycle machinery [43].

G-quadruplexes may form in telomeric DNA repeats as well as in sequences in the promoter and other regulatory regions within human and other genomes. The human genome has been surveyed for putative G4-forming sequences, and over 250,000 non-overlapping sequences have been identified [43], with many over-represented in promoter regions and in the first intron [44] of a variety of genes. Putative G4-forming regions of DNA have at least four runs of three or more consecutive guanines (G-tracts) separated by varying nucleotides that comprise the loop structures. Negative superhelicity induced by transcription can promote local unwinding of these G/C-rich duplex regions of DNA, allowing for the formation of G4s. Interestingly, a correlation has been found between the frequency of occurrence of these sequences in promoter regions and the nuclease hypersensitivity of these sites. Even though G4 formation has to compete with the stable duplex DNA form, a number of studies have shown that G4 structures can be induced under conditions which mimic the intracellular environment, and that they can have significant biological consequences [45], [46]. Crucially, G4s have recently been shown to form in cells, and to play a role in modulating transcription [47], [48], [49]. Their unique, non-B-form globular structure, and their potential to regulate the transcription of a range of oncogenes, have made G4s an attractive target for the development of novel anticancer agents. For example, a number of genes involved in the proliferation of tumour cells such as *C-myc*, *c-Kit*, *BCL2* and *K-ras* are known to contain G4-forming sequences in their promoter regions [50] and are being targeted. In a number of these, stable G4 structures have been demonstrated to form using biophysical methods, and in others (*e.g.*, *C-myc*, *c-Kit*, *K-ras*, *PDGFA* and *BCL2*) [51], [52] the molecular structures of promoter quadruplexes have been characterized by high-field NMR.

**Figure 2:**
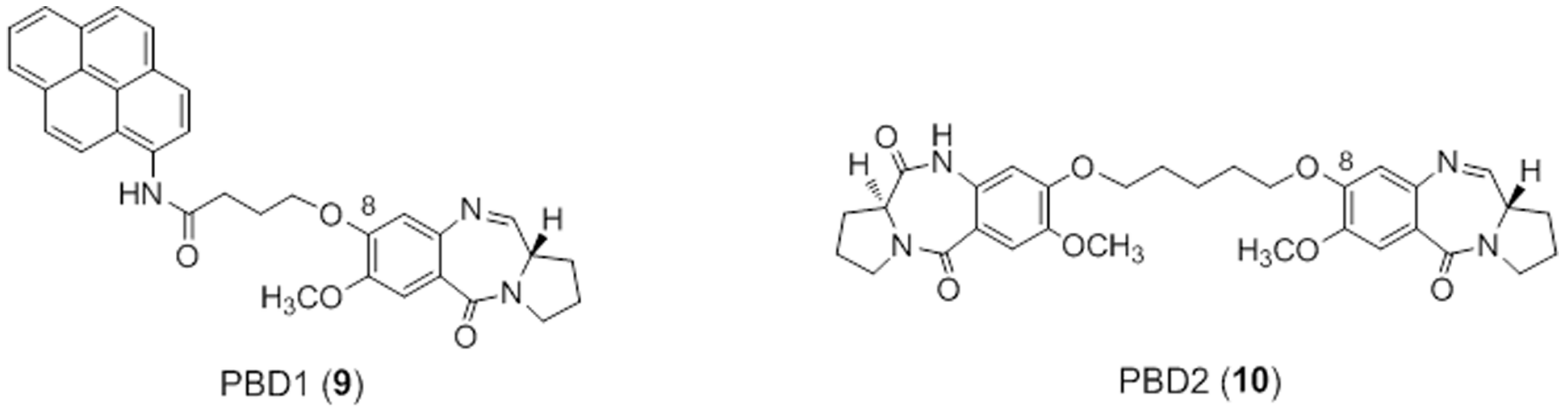
Structures of the C8-substituted PBD-Pyrene conjugate (PBD1, 9) and the PBD dimer (PBD2, 10) reported by Raju and co-workers to bind to quadruplex DNA [53].

Despite the fact that it is well-established that G-quadruplexes do not possess any B-form duplex DNA structure or minor groove environment, and that it is known that small molecules can discriminate between DNA duplex and quadruplex architectures [50], Raju and co-workers recently reported [53] that two DNA minor-groove binding PBD molecules, the C8-linked PBD-pyrene conjugate (**9**, **Figure 2**) and the mixed N10-C11/N10'-C11' imine-lactam pyrrolobenzodiazepine dimer (**10**, **Figure 2**) bind with high affinity to the G-quadruplex formed in a telomeric region of DNA from *Tetrahymena* Glaucoma. This was surprising given the extensive literature on PBD agents since the 1960s, all of which highlight the close 3-dimensional fit of these molecules in the minor groove of B-Form DNA [26], [28]. It is also known that PBDs do not bind to RNA due to the lack of a minor groove environment in this type of nucleic acid structure [54]. In addition, we have previously used PBD molecules as negative controls in screens searching for quadruplex-binding ligands (*un-reported results*).

**Figure 3:**
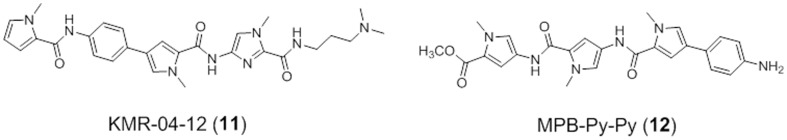
Structures of the G-quadruplex-interacting ligand KMR-04-12 (11), and the MPB-Py-Py fragment (12) identical to the C8-side chain of KMR-28-18 (5). The MPB moiety in the centre of KMR-04-12 (11) provides the molecule with a U-shaped 3-dimensional shape suitable for binding to G-quadruplex DNA.

This prompted us to carry out a detailed evaluation of the potential interaction of eight pyrrolobenzodiazepine molecules of both monomer (**1**–**5**) and dimer (**6**–**8**) types of diverse structure (**Figure 1**) with eight different G-quadruplex-forming sequences including the same *Tetrahymena* glaucoma sequence used by Raju and co-workers [53] (**Table 1**). To evaluate quadruplex *versus* duplex binding, we initially utilised a well-established FRET-based melting assay [55], [56], along with a control biaryl polyamide molecule KMR-04-12 (**11**, **Figure 3**), previously reported [55] to be highly selective for quadruplex *versus* duplex DNA. Despite evaluating the eight pyrrolobenzodiazepine compounds up to very high concentrations (*i.e.*, 5∶1 to 50∶1 ligand/DNA molar ratios), six did not show any significant stabilisation of any of the eight G-quadruplex structures, whereas GWL-78 (**4**) and KMR-28-18 (**5)** had modest stabilizing properties (*i.e.*, up to Δ*T*_m_ = 2.9 °C) at the highest concentration tested (*i.e.*, 50 µM, 50∶1 ligand/DNA molar ratio). This compared to a Δ*T*_m_ value of up to 25.1°C for the positive quadruplex-binding control molecule **11** at the same concentration and molar ratio. Conversely, when the PBDs were evaluated against duplex DNA at the same concentration and molar ratio, Δ*T*_m_ values ranged from 4.5°C for the simple PBD C8-Ester (**2**) to 28°C for the PBD dimer SJG-136 (**6**), whereas the quadruplex-binding control molecule **11** had a Δ*T*_m_ of only 1.1°C at the same concentration and molar ratio.

**Table 1:** G-Quadruplex-forming oligonucleotides used in this study.

Source Gene	Topology in K^+^ Buffer^a^	Base Pair Sequence
*F21T*	M	5′-FAM-GGGTTAGGGTTAGGGTTAGGG-TAMRA-3′
*FT2G8*	P	5′-FAM-GGGGTTGGGG-TAMRA-3′
*c-Kit1*	A	5′-FAM-d(G_3_AG_3_CGCTG_3_AG_2_AG_3_)-TAMRA-3′
*c-Kit2*	A	5′-FAM-d(G_3_CG_3_CGCGAG_3_AG_4_)-TAMRA-3′
*C-myc*	P	5′-FAM-TGGGGAGGGTGGGGAGGGTGGGGAAGG-TAMRA-3′
*K-ras*	P	5′-FAM-GGGAGGGAGGGAAGGAGGGAGGGAGGGA-TAMRA-3′
*STAT3*	P	5′-FAM-GGGCTGGGGATGGGGAGGGGG-TAMRA-3′
*BCL2*	P	5′-FAM-GGGCGCGGGAGGGAAGGGGGCGGG-TAMRA-3′
Duplex		5′-FAM-(TA)_2_GC(TA)_2_AGAT_6_TCT(TA)_2_GC(TA)_2_-TAMRA-3′

a M  =  Mixed Parallel/Anti-Parallel; P  =  Parallel; A  =  Anti-Parallel.

It is noteworthy that **4** and **5** have the longest C8-substituents of all the PBDs evaluated. Significantly, the isolated PBD core unit alone with the short C8-substituent (**2**, **Figure 1**) had no stabilising properties toward quadruplex DNA, confirming that it was the MPB-Py-Py C8-substituent itself rather than the PBD component providing the quadruplex binding properties. To investigate this further, the isolated MPB-Py-Py fragment (**12**, **Figure 3**) that comprises the C8-substituent of **5** was evaluated and found to have a Δ*T*_m_ value of up to 2.9°C at a 50∶1 ligand/DNA molar ratio. In the case of the PBD1 (**9**) and PBD2 (**10**) molecules studied by Raju and co-workers [53], it is likely that a similar phenomenon was taking place during their experiments, with the relatively bulky C8-substituents binding to the quadruplex DNA rather than the PBD components. In our study, the relative lack of interaction of the eight PBD molecules with the different quadruplex sequences was further confirmed by CD spectroscopy which failed to show any notable change in the characteristic signals after the addition of up to 10 molar equivalents of ligands. Molecular modeling studies also supported these observations.

## Materials and Methods

### Fluorescence Resonance Energy Transfer (FRET) Assay

Oligonucleotide sequences used for the FRET-based DNA thermal denaturation assays (see **Table 1**) were purchased from Eurogentec, Southampton, UK. TAMRA (6-carboxytetramethylrhodamine) and FAM (6-carboxyfluorescein) are acceptor and donor fluorophores, respectively. From 20 µM stock solutions, 400 nM solutions in FRET buffer (optimized as 50 mM potassium, 50 mM cacodylate, pH 7.4) were prepared prior to use. The oligonucleotides were annealed through heating the samples to 90°C for 10 mins followed by cooling to room temperature and storing at this temperature for 5 h. Dilutions from the initial 5 mM DMSO stock solution were performed using FRET buffer. Annealed DNA (25 µL) and sample solution (25 µL) were added to each well of a 96-well plate (MJ Research, Waltham, MA), and processed in a DNA Engine Opticon (MJ Research). Fluorescence readings were taken at intervals of 0.5°C over the range 30–100°C, with a constant temperature maintained for 30 seconds prior to each reading. Incident radiation of 450–495 nm was used, with detection at 515–545 nm. The raw data were imported into the program Origin (Version 7.0, OringinLab Corp.), and the graphs were smoothed using a 10-point running average, and then normalized. Determination of melting temperatures was based on values at the maxima of the first derivative of the smoothed melting curves using a script. The difference between the melting temperature of each sample and that of the blank (*ΔT*_m_) was used for comparative purposes.

### Circular Dichroism and Thermal Denaturation Studies

The UV & CD spectra of the oligonucleotides and oligonucleotide/ligand complexes were acquired using a Chirascan-Plus Spectrometer (Applied Photophysics Ltd, Leatherhead, UK). The UV absorbance and CD spectra were measured between 500–200 nm in a strain-free rectangular 5 or 10 mm cell. The instrument was flushed continuously with pure evaporated nitrogen throughout the measurements. Spectra were recorded using a 1 nm step size, a 1 s time-per-point and a spectral bandwidth of 1 or 2 nm. Addition of the ligands to the oligonucleotide solutions was carried out while maintaining a constant concentration of DNA. All spectra were acquired at room temperature and the buffer baseline corrected. All CD spectra were smoothed using the Savitsky-Golay method, and a window factor of 4–12 was used for better presentation.

The Dynamic Multi-mode Spectroscopy (DMS) technology provided by Applied Photophysics Ltd was employed for the thermal measurements. The CD spectra were first recorded at room temperature (20°C), then after cooling to 6°C, heating to the highest temperature (94°C), re-cooling to 6°C and finally heating back to 20°C. The melting profiles monitored at a particular wavelength were recorded during both the heating and cooling processes. The instrument was equipped with a Quantum (NorthWest, USA) TC125 Peltier unit set to change temperature from 6→94°C at a rate of 1°C/min with a 2°C step-size. The same parameters were set for the cooling process (94→6°C). A 2 s time-per-points CD measurement time scale was employed in the 400–215 nm region with a 2 nm spectral bandwidth. Temperature was measured directly using a thermocouple probe in the solutions. Melting temperatures were determined from derivative spectra produced using the Global Analysis T-Ramp software (Applied Photophysics Ltd).

### Molecular Modeling Studies

All MD simulations were performed using the AMBER 11 software package [57].

#### Ligand Preparation

The PBD structures KMR-28-18 (**5**), DRG-16 (**7**), PBD1 (**9**) and PBD2 (**10**) were constructed using the Schrödinger Maestro software, and the dihedrals of each structure were rotated to maintain the known curved nature (“isohelicity”) of the PBD molecules. AMBER *antechamber* was used to assign partial atomic charges to each ligand using the Gasteiger charging system, and missing parameters were generated for each ligand using AMBER *parmchk*. Ligands were then energy minimized using the MMFF94 energy gradient.

#### Receptor Preparation

Structural co-ordinates of the G-quadruplex receptor file were obtained from the Protein Data Bank (PDB ID: 3CDM) [58]. The coordinates were subjected to molecular mechanics minimization (1000 steps steepest descent, followed by 3000 steps of Polak-Ribiere conjugate gradient minimization with a derivative convergence of 0.05 kJ Å ^−1^ mol), followed by molecular dynamics simulation (40 ps at 300 K equilibrium using a 1.5 fs time-step). A final molecular mechanics minimization (Polak-Ribiere conjugate gradient) of the average co-ordinates of the previous MD step was then undertaken to obtain a final model of low energy for use in ligand:DNA studies [59].

#### DNA-Drug Adduct Simulation

AMBER *xleap* was used to align each ligand with the outer face of the quadruplex structure, with initial coordinate and topology files established using ff99bsc0 force field parameters [60] (for DNA) and *gaff* parameters for each ligand. AMBER *xleap* was used to automatically place 21 K^+^ counter-ions around the quadruplex structure, and a 12 Å truncated octahedral periodic water solvent box (TIP3P) and periodic boundary conditions were created for use in simulations. Initial energy minimization was undertaken with DNA restrained at a high force constant (500 kcal mol^−1^ Å^−2^), which was reduced in stages to zero. In the first stage of MD simulation, a 30 ps simulation was performed where the system was heated slowly from 0 K to 300 K using the Langevin thermostat (and a collision frequency of 1.0 ps^−1^). Following this, an equilibration step was undertaken over 100 ps with constant pressure and no restraints. Production dynamics simulations were then run over a time-scale of 10 ns (constant volume, 300 K temperature) with a 2 fs time step. The SHAKE algorithm was applied to restrain hydrogen atoms, thereby removing the highest frequency oscillation and permitting the use of a 2 fs time-step, with all simulations undertaken using AMBER *pmemd*. Molecular dynamics simulations were visualised using VMD [61], and models were created using Chimera [62].

#### Free Energy of Binding Calculations

Binding free energy was calculated using the following equation:
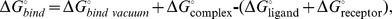
where Δ*G*°*_bind_* was determined by solving the linearized Poisson-Boltzmann equation [63]:
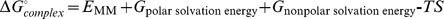



Solvation energies (both polar and non-polar) were considered, with *E*_MM_ corresponding to internal, electrostatic and van der Waal interactions, and *S* to solute entropy.

The final binding energy was represented using the equation:
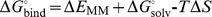



Entropic calculations were not undertaken as they are computationally expensive and are prone to introducing significant errors into the calculation of free energy of binding values. Furthermore, due to the fact that similar entropic states (*i.e.*, identical receptors and structurally similar ligands) were analyzed, computationally expensive entropic calculations would have added little value to the free energy of binding results. One hundred snapshots of the MD simulations were taken at equal intervals over the 10 ns duration, and molecular mechanics (MM) calculations were performed using *pbsa* [64].

## Results and Discussion

The initial experiments involved measurement of the interaction of PBDs **1**–**8** (**Figure** **1**) and the quadruplex-binding control molecule **11** (**Figure 3**) with the eight quadruplex-forming DNA sequences and the control DNA duplex shown in **Table 1** using the Fluorescence Resonance Energy Transfer (FRET) thermal denaturation assay described in the *Materials and Methods* section. FRET assays are widely used to determine melting temperatures of different DNA sequences and ligand-DNA complexes [65], [66], and the FRET phenomenon has been extensively used to study the stability of nucleic acid structures and, in particular, G-quadruplex DNA [65]. In the FRET-based G-quadruplex melting assay used in this study, 6-carboxyfluorescein (FAM) and 6-carboxytetramethylrhodamine (TAMRA) were used as donor and acceptor FRET moieties attached to the 5′- and 3′- ends of the DNA, respectively, via 6-carbon linkers [66]. The FAM/TAMRA fluorophore pair has a Forster radius (Ro) of approximately 5 nm, and when the labeled sequences form G-quadruplexes, the two are located close enough to allow FRET to occur efficiently. Application of external energy in the form of heat unfolds the quadruplex causing the fluorophores to separate from each other, thus reducing the FRET efficiency and generating a signal through a consequent increase in the intensity of radiation emitted by the donor.

The results (**Table 2**) confirm that the PBD monomers without heterocycle-containing C8-substituents (**1**–**3**), all failed to stabilise the eight G-quadruplex sequences even at a molar ratio of 50∶1 (Ligand:G-quadruplex - 10 uM:0.2 uM and 50 µM:1 µM). This was anticipated, as the molecules are not planar and contain no appreciable π-ring systems (apart from the PBD A-ring) that can interact with the quadruplex structure, a known means to enhance ligand/quadruplex affinity [67], [68]. On the other hand, the PBD-C8 conjugates with π-ring-containing C8-substituents (**4** and **5**) showed negligible stabilisation at a molar ratio of ligand/quadruplex of up to 5∶1, but a modest increase in melting temperatures (*i.e.*, up to 2.4°C) of almost all the G-quadruplexes at a 25∶1 ratio (Ligand:G-quadruplex - 5 uM:0.2 uM), increasing marginally to up to 2.9°C at a 50∶1 molar ratio (Ligand:G-quadruplex - 10 uM:0.2 uM and 50 µM:1 µM). This suggested that, for these molecules, the stabilisation was due to the heterocyclic rings attached to the C8-positon, particularly as the shorter C8-substituent of **4** led to a lower enhancement in *T*_m_ than the longer side chain of **5**. To probe this further, we evaluated the dipyrrole-MPB moiety **12** (**Figure 3**), identical in structure to the C8-substituent of KMR-28-18 (**5**), at the same concentrations as used for the PBD-C8 conjugates. Interestingly, it produced an almost identical interaction profile, stabilising all of the different G-quadruplex sequences by up to 2.1°C at a 50∶1 molar ratio (Ligand:G-quadruplex - 10 uM:0.2 uM and 50 µM:1 µM). This result, coupled with the fact that the simple PBD monomers **1**–**3** had no quadruplex stabilising activity of their own even at the highest concentration and molar ratio, confirmed that PBD structures themselves are not capable of stabilizing DNA quadruplex.

**Table 2:** FRET-based DNA thermal denaturation assay results (Δ*T*_m_ values) for the interaction of the PBD monomers (**1**–**5**) and dimers (**6**–**8**) (**Figure 1**) with eight different G-quadruplex-forming DNA sequences and a duplex DNA control (**Table 1**).

Ligand	Ligand Conc. [µM] (Ligand:G4)	ΔTm [°C]^a^								
		*F21T*	*FT2G8*	*c-Kit1*	*c-Kit2*	*C-myc*	*K-ras*	*STAT3*	*BCL2*	Duplex
**Anthramycin (1)**	**50 (50∶1)** b	0	0	0	0	0	0	0	0	18
	**10 (50∶1)**	0	0	0	0	0	0	0	0	14
	**5 (25∶1)**	0	0	0	0	0	0	0	0	12
	**1 (5∶1)**	0	0	0	0	0	0	0	0	11
**PBD-C8-Ester (2)**	**50 (50∶1)** b	0	0	0	0	0	0	0	0	4.7
	**10 (50∶1)**	0	0	0	0	0	0	0	0	4.5
	**5 (25∶1)**	0	0	0	0	0	0	0	0	4.0
	**1 (5∶1)**	0	0	0	0	0	0	0	0	2.3
**PBD-C8-Morpholine (3)**	**50 (50∶1)** b	0	0	0	0	0	0	0	0	7.1
	**10 (50∶1)**	0	0	0	0	0	0	0	0	6.6
	**5 (25∶1)**	0	0	0	0	0	0	0	0	7.5
	**1 (5∶1)**	0	0	0	0	0	0	0	0	6
**GWL-78 (4)**	**50 (50∶1)** b	1.5	1.5	2.2	2.0	0	2.3	2.0	2.0	23
	**10 (50∶1)**	1.2	1.2	2.0	2.1	0	2.1	1.8	1.9	24
	**5 (25∶1)**	1.1	0.6	1.2	1.4	0	1.1	1.1	0.8	19
	**1 (5∶1)**	0	0	0	0	0	0	0	0	17
**KMR-28-18 (5)**	**50 (50∶1)** b	2.5	2.0	2.3	2.7	1	2.2	2.0	2.1	18
	**10 (50∶1)**	2.8	2.1	2.5	2.9	1	2.4	2.2	2.4	20
	**5 (25∶1)**	1.0	1.2	2.2	2.4	0.5	2.1	2.0	1.8	18
	**1 (5∶1)**	0.5	0.5	0.5	0.5	0	0.5	0	0.5	14
**SJG-136 (6)**	**50 (50∶1)** b	0.9	0.0	0.6	0.8	0	1.4	1.1	0.8	23
	**10 (50∶1)**	0.8	0.0	0.8	1.0	0	1.1	1.2	0.8	28
	**5 (25∶1)**	0.6	0.5	0.7	0.7	0	0.8	0.7	0.5	22
	**1 (5∶1)**	0	0	0	0	0	0	0	0	17
**DRG-16 (7)**	**10 (50∶1)**	1.0	0.7	0.9	0.8	0	1.5	1.7	1.1	28
	**5 (25∶1)**	0.8	0.5	0.6	0.6	0	0.8	0.6	0.8	21
	**1 (5∶1)**	0	0	0	0	0	0	0	0	18
**ELB-21 (8)**	**50 (50∶1)** b	0.8	0.7	0.6	0.8	0	0.8	0.9	0.8	25
	**5 (25∶1)**	0.6	0.6	0.5	0.6	0	0.6	0.5	0.8	24
	**1 (5∶1)**	0	0	0	0	0	0	0	0	17
**KMR-04-12 (11)**	**50 (50∶1)** b	26.5	18.5	15.3	14.5	5.5	14.2	12.5	12.0	1.5
	**10 (50∶1)**	25.1	17.1	14.5	15.6	4.5	13.7	11.2	12.3	1.1
	**5 (25∶1)**	17.5	12.5	10.6	8.5	2.3	11.3	8.6	10.5	0.7
	**1 (5∶1)**	8.8	4.6	6.1	3.2	2.1	7.7	5.7	6.2	0
**MPB-Py-Py (12)**	**50 (50∶1)** b	2.0	2.1	2.0	2.2	0	2.1	2.2	1.8	1.4
	**10 (50∶1)**	1.8	1.6	1.9	2.1	0	1.1	1.8	1.9	1.2
	**5 (25∶1)**	0.8	1.1	1.5	1.6	0	1.5	1.6	1.8	1.0
	**1 (5∶1)**	0	0.5	0	0.5	0	0.5	0	0.5	0

KMR-04-12 (**11**, **Figure 3**) was included as a positive quadruplex-binding control molecule, and the MPB-Py-Py fragment **12** (**Figure 3**) was evaluated to represent the isolated C8-substituent of the PBD C8-Conjugate **5** (**Figure 1**).

a ESDS±0.5°C

b Concentration of DNA was 1 µM in these experiments; in all other cases it was 200 nM. Differences in Δ*T*_m_ values for duplex DNA at the highest ligand concentration of 50 µM (50∶1, ligand:G4) are thought to be due to solubility or aggregation issues at these artificially high concentrations of ligand and DNA.

**Figure 4:**
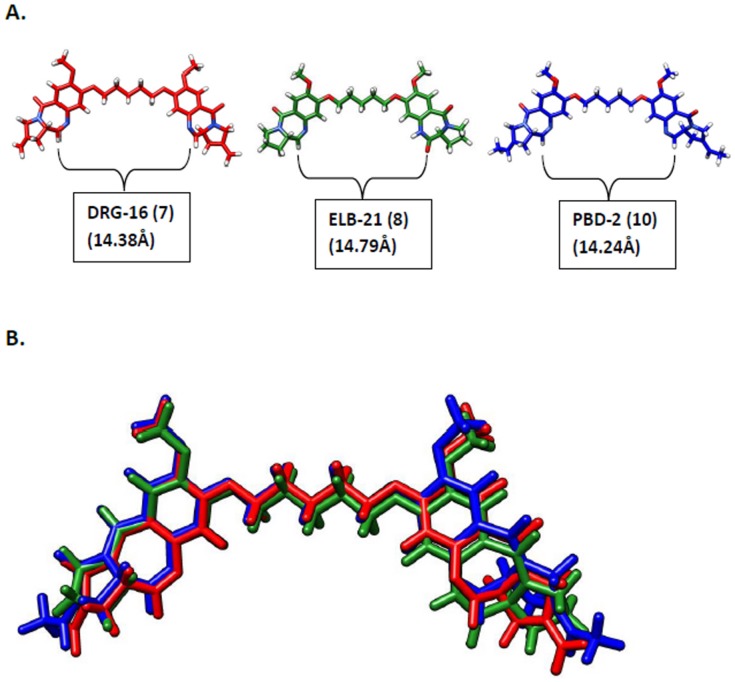
**A**, Comparison of length and 3-dimensional shape between the PBD dimer (PBD2, **10**) used in the G-quadruplex studies reported by Raju and co-workers [53] and the PBD dimers **7** and **8** used in this study; **B**, Overlay of the three PBD dimers (**7**, **8** and **10**) confirming their superimposability.

Next, we studied the PBD dimers **6**–**8** for their ability to interact with and stabilise the G-quadruplex sequences. PBD dimers **7** and **8** are similar to the PBD2 (**10**) molecule (**Figure 2**) studied by Raju and co-workers in having a C8/C8'-dioxypentenyl linker joining the two A-rings of the PBD units. Although they contain N10-C11/N10'-C11' imine moieties rather than the mixed imine/dilactam functionalities present in PBD2 (**10**) at these positions, this does not affect their length or 3-dimensional shape (see **Figure 4**). However, no stabilisation was observed for any of the quadruplex-forming sequences at molar ratios of up to 10∶1 (ligand:G-quadruplex). The shorter PBD dimer SJG-136 (**6**) that contains a C8/C8'-dioxypropenyl linker was similarly non-interactive. A modest increase in melting temperature was observed for all three PBD dimers (*i.e.*, up to 1.7°C) at the higher 50∶1 ratio (Ligand:G-quadruplex - 10 uM:0.2 uM), most likely due to a non-selective interaction with the loop sequences where the dimers may be able to form hairpin-like structures around the DNA bases at such high concentrations.

**Figure 5:**
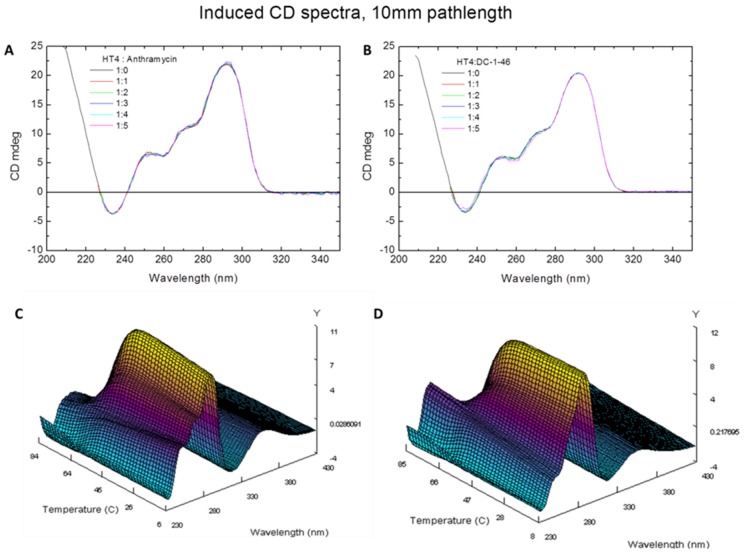
**A** & **B**, CD titration of anthramycin (**1**) and the C8-PBD-morpholine (**3**), respectively, with the telomeric G-quadruplex sequence (HT4) demonstrating a lack of interaction; **C** & **D**, CD melting profile of anthramycin:HT4 and C8-PBD-morpholine (**3**):HT4, respectively. For both molecules there was no change in CD melting temperature compared to the HT4 DNA alone.

**Figure 6:**
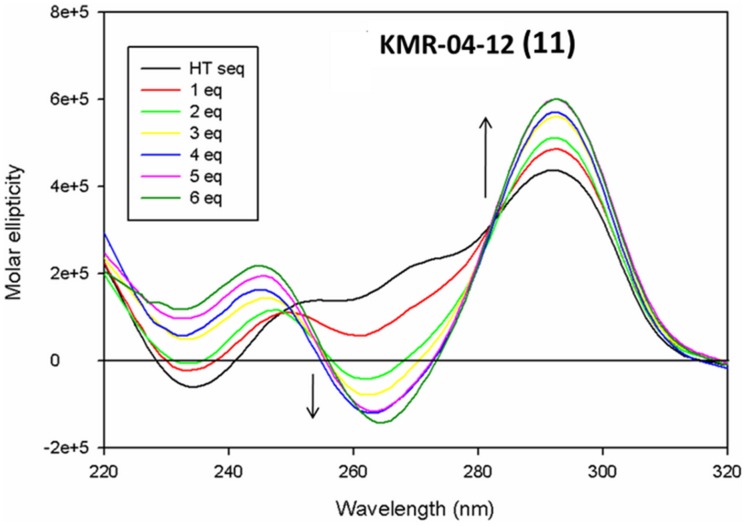
CD spectrum of a 5 µM solution of telomeric G-quadruplex with 0–6 equivalents of KMR-04-12 (11), showing dose-dependent enhancement of the CD signals.

To further confirm the lack of interaction of PBD monomers and dimers with G-quadruplex sequences, CD titration and CD melting studies were carried out with the PBD monomer anthramycin (**1**) and the C8-linked PBD-morpholine conjugate **3** with the human telomeric G-quadruplex sequence HT4. This quadruplex was selected as it is one of the most studied structures, and a number of CD titration experiments with G-quadruplex-binding ligands have been reported [45], [51], [56], [67], [69]. The CD spectrum of the HT4 sequence showed the characteristic presence of mixed parallel and antiparallel structures with positive peaks around 295 nm and a negative peak around 240 nm (**Figure 5**). Neither **1** or **3** produced any notable changes in the CD signals at the highest ratio evaluated (5∶1; ligand:HT4). Furthermore, no change in melting temperatures in the presence or absence of the ligands was observed in the CD melting experiment. On the other hand, for the known quadruplex-binding ligand KMR-04-12 (**11**, **Figure 3**), we observed a concentration-dependent enhancement of the major positive peak at 295 nm, a concentration dependent appearance of a major negative peak around 260 nm, and a concentration dependent disappearance of the negative peak around 240 nm, all confirming association of the ligand with the telomeric G-quadruplex structure (**Figure 6**).

Next, a detailed molecular modelling study was carried out to further confirm the lack of interaction of pyrrolobenzodiazepine dimers with G-quadruplex DNA. For this study we selected the PBD dimer PBD2 (**10**) reported by Raju and co-workers [53], and the two structurally similar PBD dimers **7** and **8**, both of which have been previously reported in the literature [1], [21] and have similar length and 3-dimensional structure (**Figure 4A**). Furthermore, an overlay of **7**, **8** and **10** shows that each one occupies similar 3-dimensional space and should cover a near-identical span of DNA bases (**Figure 4B**). As the shape and curvature of each structure is identical, PBD2 (**10**) would be expected to interact with duplex rather than quadruplex DNA in a similar manner to **7** and **8**. Although we did not have access to PBD2 (**10**), the shape similarity between these three PBD dimers should enable extrapolation of the experimental results obtained to **10**. As we did not observe any notable stabilisation of the G-quadruplex sequences (including the *Tetrahymena* G-quadruplex) by either **7** or **8**, it would appear unlikely that PBD2 (**10**) could provide the strong G-quadruplex stabilisation reported by Raju and co-workers [53].

**Figure 7:**
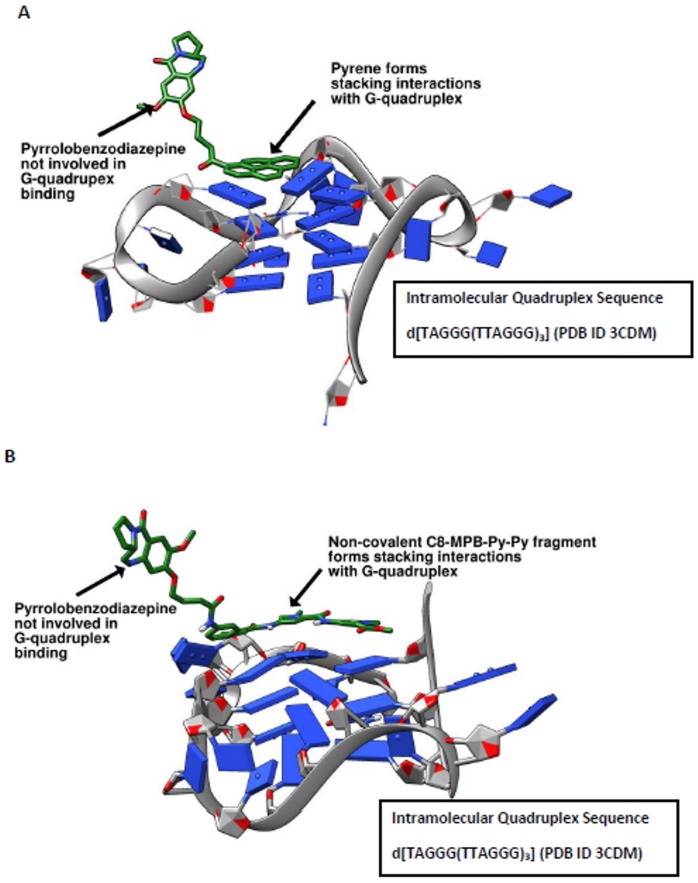
**A**, Snapshot of a MD simulation of the PBD-Pyrene conjugate (PBD1, **9**) interacting with the telomeric G-quadruplex, showing the the C8-pyrene component stacking with the G-quartet of the telomeric quadruplex, and the PBD component pointing upwards in an orthogonal orientation to the pyrene moiety; **B**, Snapshot of a MD simulation of the C8-PBD conjugate KMR-28-18 (**5**) interacting with the telomeric G-quadruplex, showing a similar interaction of the C8-side chain with the quadruplex DNA rather than the PBD moiety itself.

Finally, we carried out molecular dynamics studies on the C8-pyrene conjugate (PBD1, **9**, **Figure 2**) reported by Raju and co-workers, and the C8-MPB-Py-Py conjugate (**5**, **Figure 1**) used in our study, to elucidate a possible mechanism of binding of these molecules to quadruplex DNA. In the FRET-DNA melting study, **5** provided modest G-quadruplex stabilisation of up to 2.9°C at a molar ratio of 50∶1 (ligand:DNA). The crystal structure of a telomeric G-quadruplex (PDB ID: 3CDM) was taken as a starting point to study plausible interactions with **5** and **9**. In both cases, the MD simulations suggested that quadruplex binding was mediated exclusively by the C8-linked components and not by the PBD ring structure itself. In the case of **9**, when it was placed over the G-quartet, the planar pyrene fragment appeared to be ideally shaped for interaction with the G-quadruplex structure. In the initial stages of the simulation, the pyrene moiety interacted in a non-covalent manner with the G-quadruplex sequence, with the pyrrolobenzodiazepine unit pointing upwards in an orthogonal position to the pyrene moiety (**Figure 7a**). As the simulation progressed, the PBD fragment was gradually pulled further towards the quadruplex structure, but did not participate in any significant interactions with the DNA. Further simulations were undertaken starting with the PBD placed over the G-quadruplex and the pyrene oriented at an angle orthogonal to the G quartet. However, in these simulations the PBD shifted away from the quadruplex structure and was replaced by the pyrene which then stabilised the G-quadruplex to a much greater extent. In the case of the PBD-MPB conjugate **5**, which exhibited moderate G-quadruplex stabilisation, simulations of the ligand interacting with the quadruplex produced similar results to those for the PBD-Pyrene (**9**), with the non-covalent moiety (*i.e.*, the C8-MPB-Py-Py fragment) interacting with the G-quadruplex structure while the PBD remained outside of the plane of the DNA. These observations were further supported by free energy of binding calculations conducted during simulations of the interaction of the C8-Pyrene conjugate (**9**) with the G-quadruplex compared to similar calculations for interaction of the pyrene fragment alone with the quadruplex. These calculations gave a more favourable free energy of binding for the pyrene fragment alone (−19.50 kcal/mol) compared to the C8-Pyrene PBD conjugate (**9**) (−17.94 kcal/mol), suggesting that the presence of the PBD fragment may actually reduce DNA interactivity rather than enhance it. Taken together, these simulations and energy calculations unequivocally supported our view that it is the non-covalent C8-moieties of the ligands (*i.e.*, the MPB-Py-Py and pyrene fragments of **5** and **9**, respectively) that direct and facilitate quadruplex binding rather than the PBD entities themselves, which have no affinity for quadruplex DNA.

## Conclusion

Despite the report by Raju and co-workers [53] that PBD molecules can stabilise quadruplex DNA structures, we can find no evidence to substantiate this claim based on FRET thermal denaturation, CD studies, molecular dynamics simulations and free energy of binding calculations of the potential interaction of eight PBD molecules of diverse structure with eight different DNA quadruplex types. For some PBD-containing molecules we did observe moderate binding at high molar ratios of ligand/DNA, but this only occurred to a significant extent for PBD molecules with large C8-substituents (*e.g.*, **4** and **5**). We note that both the PBD1 (**9**) and PBD2 (**10**) molecules studied by Raju and co-workers had large C8-substituents, and it is likely that they were observing the same non-specific interactions of the C8-substituents with the quadruplex DNA rather than the PBD moieties themselves.

We also note that, in their recent report in *this journal* [53], Raju and co-workers state that “PBDs were previously reported to possess planar structure and intercalate to DNA”, referring to work published by Hopkins and co-workers [70] and Kraus and Selvakumar [71]. In fact, since the discovery of the first PBD (anthramycin) in the early 1960s [6], there have been no reports of this family of agents interacting with DNA by intercalation, and it is widely accepted that they are not planar due to their chiral C11a(*S*)-position and interact in the minor groove due to their inherent isohelicity with the DNA helix.

In summary, we report here experimental evidence that PBD molecules do not interact to any significant extent with quadruplex DNA structures. It is important that this is clear in the literature, as two PBD-based agents are presently in clinical trials and others are in late-stage pre-clinical development, and so the mechanism of action of these agents will be under close scrutiny in the future.
